# Evidence of P3a During Sleep, a Process Associated With Intrusions Into Consciousness in the Waking State

**DOI:** 10.3389/fnins.2018.01028

**Published:** 2019-01-10

**Authors:** Paniz Tavakoli, Allyson Dale, Addo Boafo, Kenneth Campbell

**Affiliations:** ^1^Children’s Hospital of Eastern Ontario, Ottawa, ON, Canada; ^2^School of Psychology, University of Ottawa, Ottawa, ON, Canada; ^3^Department of Psychiatry, University of Ottawa, Ottawa, ON, Canada

**Keywords:** conscious awareness, sleep, gating, event-related potentials, P3a, multi-feature paradigm

## Abstract

The present study examines processes associated with intrusions into consciousness during an unconscious state, natural sleep. The definition of sleep is still much debated. Almost all researchers agree that sleep onset represents a gradual loss of consciousness of the external environment. For sleep to be beneficial, it needs to remain as undisturbed as possible. Nevertheless, unlike other unconsciousness states, sleep is reversible. For purposes of survival, it is critical that the sleeper be able to “detect” and perhaps become conscious of highly relevant biological or personal information. Therefore, even in sleep, the brain must decide whether a new incoming stimulus is relevant and if so, may require an arousal to wakefulness, or whether it is irrelevant and can be gated to prevent disruption of sleep. Event-related potentials (ERPs) were used to measure the extent processing of auditory stimuli some of which elicited an ERP component, the P3a, in the waking state. The P3a is associated with processes resulting in the interruption of frontal central executive, leading to conscious awareness. Very little research has focused on the occurrence of the P3a during sleep. A multi-feature paradigm was used to examine the processing of a frequently occurring “standard” stimulus and six rarely occurring different “deviant” stimuli during wakefulness, NREM, and REM sleep. A P3a was elicited by novel environmental sounds and white noise bursts in the waking state, replicating previous studies. Other deviant stimuli (changes in pitch, intensity, duration) failed to do so. The ERPs indicated that processing of the stimuli that did not elicit a P3a in wakefulness were much inhibited during both NREM and REM sleep. Surprisingly, those deviants that did elicit a P3a in wakefulness continued to do so in stage N2 and REM sleep. The subject did not, however, awaken. These results suggest processes leading to consciousness in wakefulness may still remain active during sleep possibly allowing subjects to act on potentially highly relevant input. This may also explain how sleep can be reversed if the stimulus input is sufficiently critical.

## Introduction

Natural sleep is a period of profound unconsciousness. Unlike other unconscious states, natural sleep is, however, rapidly reversible. For purposes of survival, the sleeper must have the ability to detect highly relevant external information, whether biological or personal, and if necessary, to awaken to a conscious state and take appropriate action. Nevertheless, for sleep to be beneficial, all but the most relevant external stimulation is inhibited. Therefore, even in sleep, the brain must decide whether incoming stimuli require further processing and possible arousal to wakefulness, or whether they can be inhibited (Campbell and Colrain, 2002). The present study examines processes associated with intrusions into consciousness during natural sleep.

Sleep is not a uniform state. It consists of a series of sub-stages that can be divided into non-REM (NREM; stages N2 and N3) and REM. The processing of external stimulus input can be differentially affected during the various stages of sleep (for reviews, see Campbell and Colrain, 2002; Massimini et al., 2009; Scammell et al., 2017). A major problem with examining the extent of information processing during sleep is that the subject cannot signal awareness of the external stimuli. In the present study, event-related potentials (ERPs) were used to measure the extent of information processing in the brain in the absence of overt behavioral responses (for reviews, see Colrain and Campbell, 2007; Koch et al., 2016; Dykstra et al., 2017). ERPs consist of a series of negative or positive components thought to reflect different stages of information processing. Some of these components can be elicited independent of attention and consciousness while others are highly affected by whether the subject attends to the stimulus input and becomes conscious of it.

The ability to detect acoustic changes in the environment is particularly important. The detection of acoustic change and possible subsequent intrusions into consciousness is often studied using an auditory oddball paradigm, consisting of a frequently occurring “standard” stimulus and rarely occurring “deviants.” The presentation of the standard stimulus elicits an obligatory negativity peaking at about 100 ms, N1, and a positivity peaking at about 200 ms, P2. The N1 and P2 are mainly associated with sensory processing of the auditory stimuli. They increase in amplitude to loud and infrequently presented auditory stimuli. The N1 gradually decreases in amplitude during sleep onset until it reaches near baseline levels during stage N2 sleep. On the other hand, P2 has been observed to increase in amplitude during sleep (Harsh et al., 1994; Cote et al., 2001; Campbell and Colrain, 2002; Crowley et al., 2002).

The deviant stimulus also elicits the N1 and P2, but in addition elicits a negative component, the mismatch negativity (MMN) occurring at about 100–200 ms after stimulus onset (Näätänen, 1990, 1992). It is maximum over fronto-central areas of the scalp inverting in polarity at the mastoids. The MMN is thought to reflect the automatic detection of acoustic change. Certain types of deviants will also elicit a larger N1 than the standard. For example, a deviant that represents an increase in intensity from the standard will elicit a larger N1 and MMN. This composite negativity following this deviant represents the spatial and temporal summation of the N1+MMN. As a result, this negativity is often called a deviant-related negativity (DRN). In this article, the negativity that is elicited by the deviants will be described as a DRN.

Results from studies examining the DRN during sleep have not always been consistent. Many studies have failed to observe a distinct DRN during NREM (Nielsen-Bohlman et al., 1991; Niiyama et al., 1994; Winter et al., 1995; Loewy et al., 1996; Nordby et al., 1996; Loewy et al., 2000; Nashida et al., 2000; Macdonald et al., 2008; Sculthorpe et al., 2009; Strauss et al., 2015) and REM sleep (Niiyama et al., 1994; Sallinen et al., 1996; Loewy et al., 2000; Strauss et al., 2015). On the other hand, some authors have reported that a DRN can be elicited by many different types of deviants during NREM (Sabri et al., 2000, 2003; Sabri and Campbell, 2005; Ruby et al., 2008) and REM sleep (Loewy et al., 1996; Atienza et al., 1997, 2000; Nashida et al., 2000; Atienza and Cantero, 2001; Cote et al., 2001; Sabri and Campbell, 2005; Macdonald et al., 2008; Ruby et al., 2008; Sculthorpe et al., 2009), although with reduced amplitudes compared to the waking state. It is possible that the nature or extent of the deviance change (frequency, intensity, duration) could account for these differences.

The output of the change detection system is claimed to vary directly with the extent of change. In the waking state, if the extent of change is large enough, an involuntary switch of attention from the demands of ongoing cognitive tasks and to the unattended auditory input may occur. This involuntary capture of attention or “intrusion into consciousness” is associated with a later positivity, P3a (Escera et al., 1998). The P3a occurs at about 200–300 ms following stimulus onset and is largest over centro-frontal areas of the scalp. There is much debate about the extent to which the P3a reflects actual consciousness of the potentially highly relevant auditory input. Some authors suggest that it reflects a precursory process occurring prior to the switch of cognitive resources to the auditory channel that may only then subsequently lead to conscious awareness (Wetzel et al., 2013; Parmentier, 2014). There is, however, general agreement that the P3a does reflect processes associated with the involuntary capture of attention. In the waking state, while any perceptible change in stimulation will elicit a DRN, only a small number will also elicit a P3a.

Few studies have examined the P3a during sleep. Most MMN/DRN studies did not report a P3a during sleep, possibly because the extent of deviance was not sufficiently large. Cote and Campbell (1999) observed a frontal positivity at about 250 ms to rarely occurring deviant stimuli that represented a large increase in intensity (90 dB SPL) from the standard (70 dB SPL) during REM sleep. The latency and scalp distribution of this positivity did correspond to that of the P3a. This positivity was followed by a second large centro-parietal positivity, the P3b, occurring at about 320 ms, similar to that which was recorded in wakefulness. Macdonald et al. (2008) also observed a P3a-like positivity during REM sleep, occurring at about 270 ms, following a 10 dB increase in intensity from the 80 dB SPL standard. They did not observe a P3a following a 20 dB decrease in intensity deviant during REM. The P3a can also be elicited by deviants that are not obtrusive. Ruby et al. (2008) employed a duration deviant and reported the presence of both a P3a-like positivity and a later P3b-like positivity occurring at about 200 and 300 ms, respectively, in both stage N2 and REM. Tavakoli et al. (2018) recorded the P3a during stage N1 and the first 30 min of stage N2 sleep. They observed a significant P3a-like positivity at about 240 ms during early stage N2 sleep following their environmental sound deviants. A P3a-like positivity was also observed following their white noise deviant during this stage at about 220 ms, although its amplitude did not attain significance.

Most studies employ oddball sequences. A problem with this paradigm is that only one or perhaps two deviants can be presented within the sequence. A reason for the somewhat contradictory findings during sleep may be the use of different deviants across studies. The oddball paradigm does not easily allow for the presentation of many types of deviants, limiting the extent to which the effect of deviants can be compared in a single study. On the other hand, a more recent multi-feature paradigm (Näätänen et al., 2004), as its name implies, allows for the presentation of several deviants each representing a change of a different feature of the standard. The present study employs a multi-feature paradigm used by Tavakoli and Campbell (2016) and Tavakoli et al. (2018) to examine those deviants most likely to elicit a P3a during sleep. In the multi-feature paradigm, six different deviant stimuli were presented in a single auditory sequence, consisting of an alternating standard-deviant pattern. The DRN and P3a that are elicited within a multi-feature paradigm are very similar to those elicited within the traditional oddball paradigm (Näätänen et al., 2004; Tavakoli and Campbell, 2016). Previous studies have indicated that a change in stimulus duration or an increase in stimulus intensity might elicit a P3a-like response during sleep (Macdonald et al., 2008; Ruby et al., 2008), although they did not do so when presented within a multi-feature paradigm in fully awake young adults (Tavakoli and Campbell, 2016). The determination of what stimulus features are deemed to be so potentially relevant to warrant an interrupt of executive functions may differ between the waking and sleep states. White noise and environmental sounds do elicit a large P3a in waking subjects but have not been presented as deviants in previous sleep studies. To control for the possibility that any stimulus change might elicit a P3a within sleep, deviants representing a change in frequency and a decrease in intensity were also embedded within the multi-feature paradigm. The multi-feature paradigm was presented passively to subjects during the waking state while the subject’s attention was engaged in watching a silent, subtitled movie, and subsequently within the different stages of all-night sleep.

## Materials and Methods

### Subjects

Sixteen self-reported good sleepers (14 women) between the ages of 20–28 years (mean = 23.12 years, SD = 2.68 years) spent a single night in the sleep laboratory. All subjects reported normal sleep time to be between 23:00 and 24:00. None reported any history of hearing, neurological, or sleep disorders. Written informed consent was obtained prior to the start of the study and subjects received an honorarium for their participation. Subjects refrained from caffeine and alcohol in the 24 h prior to the start of the study. The study was conducted according to the Canadian Tri-Council guidelines (Medical, Natural, and Social Sciences) on ethical conduct involving human subjects.

### Physiological Recordings

EEG and electrooculography (EOG) activity were recorded using Grass gold-cup electrodes, filled with electrolytic paste, and affixed to the skin by surgical tape and to the scalp by gauze. Brain Products Recorder software and BrainAmp amplifiers were used for the EEG recording. The EEG was recorded from 18 electrodes across frontal, central, parietal, and occipital sites (FP1, FP2, FT9, FT10, F3, Fz, F4, FC3, FCz, FC4, C3, Cz, C4, P3, Pz, P4, O1, O2) placed according to the 10/10 system of electrode placement. Two additional electrodes were placed on the left and right mastoids (M1 and M2). A vertical EOG was recorded from electrodes placed at the supra-orbital and infra-orbital ridges of the left eye. A horizontal EOG was recorded from electrodes placed at the outer canthus of each eye. A defining characteristic of the MMN/DRN is that it inverts in polarity at the mastoids when a nose reference is used. For this reason, the tip of the nose served as a reference for all channels, including the EOG channels. Inter-electrode impedances were kept below 5 kΩ. The high frequency cut-off filter was set at 75 Hz and the time constant was set at 2 s (i.e., a low-frequency cut-off filter of 0.08 Hz). The physiological data were digitized continuously at a 500 Hz sampling rate.

### Procedure and Stimuli

**FIGURE 1:**
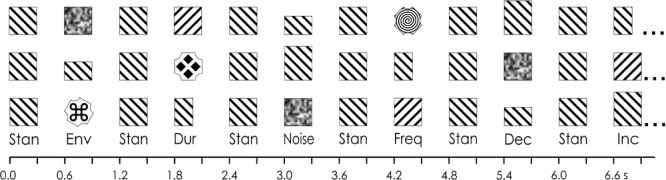
Multi-feature paradigm. In the example, in the first line, 12 stimuli are presented in an array. The sequence begins with a standard (“Stan”) and then alternating with the standards: a white noise (“Noise”) deviant, a frequency (“Freq”) deviant, a decrement (“Dec”) deviant, an environmental sound (“Env”) deviant, an increment (“Inc”) deviant, and a duration (“Dur”) deviant. In the second line, the six deviants are presented again but in a different randomized order. Note that a different environmental sound was presented throughout the sequence. Image adapted from Tavakoli and Campbell (2016).

Auditory stimuli were presented monaurally to the right ear using EAR 3A insert earphones. Ear of presentation has been shown to have very little effect on either the DRN or P3a (Grimm et al., 2008). The subject was thus able to sleep on the side where the earphone was not inserted. A multi-feature auditory paradigm was presented. The general stimulus procedure is illustrated in Figure 1. It consisted of six deviant stimuli presented in the same sequence so that every other tone was an 80 dB SPL 1,000 Hz “standard” tone burst (*p* = 0.5) and every other was one of six deviants (each with a *p* = 0.083). Thus, the standards and deviants alternated. Deviants in the multi-feature sequence included (a) a 90 dB SPL “increment” pure tone, (b) a 60 dB SPL “decrement” pure tone, (c) an 80 dB peak SPL white noise burst, (d) different environmental sounds (with an average intensity of 80 dB SPL), (e) a higher frequency, 1,100 Hz, pure tone, and (f) a shorter duration, 100 ms, pure tone. The order of presentation of the deviants were pseudo-randomized so that in an array of six deviants, each deviant was presented only once, and that the same deviant was never presented two times in a row. A different environmental sound was presented on each trial so that none of the environmental sounds were repeated. The features of the environmental sounds have been described in detail by Fabiani et al. (1996). They included animal, bird, human vocalizations, musical instruments, environmental, video-game sounds, and mechanically produced sounds. Their duration was, however, manipulated to be 200 ms. The first 10 tones in the sequence consisted of only standards in order to establish a memory trace for the standard stimulus. All stimuli had a duration of 200 ms and a rise-and-fall time of 5 ms, with the exception of the duration deviant. Table 1 lists the properties of the various auditory stimuli. The stimulus onset asynchrony (SOA; onset-to-onset) was 600 ms. A total of 932 stimuli were presented in a single sequence, consisting of 470 trials of standards and 77 trials of each deviant, lasting 9.5 min.

**Table 1:** The intensity, frequency, duration, and probability of the standard stimulus and the six deviant stimuli in the multi-feature paradigm.

Stimulus type	Intensity	Frequency	Duration (ms)	Probability
Standard	80 dB SPL	1,000 Hz	200	0.50
**Deviants**				
Frequency	80 dB SPL	*1,100 Hz*	200	0.08
Increment	*90 dB SPL*	1,000 Hz	200	0.08
Decrement	*60 dB SPL*	1,000 Hz	200	0.08
Duration	80 dB SPL	1,000 Hz	*100*	0.08
White noise	*∼80 dB SPL*	*Random*	200	0.08
Environmental sounds	*∼80 dB SPL*	*Mixed*	200	0.08

*Information in italics represents the feature of the deviant that has been changed.*

The waking data were collected between 22:00 and 23:00 while subjects were seated in a sound-attenuated room and asked to watch a silent, subtitled movie of their choice and thus to ignore the auditory stimuli. Three blocks of auditory stimuli were presented during wakefulness. A brief break was provided between blocks. Waking testing lasted for approximately 40 min, including break periods. Subjects were then permitted to fall asleep. Once subjects entered stage N2 sleep (verified by the presence of K-complexes and spindles), auditory stimulation was commenced and continued throughout the whole night. Again, stimuli were presented in a 9.5 min block. A brief 5–10 min silent period was provided between blocks. Sleep stages were classified in real-time by an experienced sleep researcher using standard scoring procedures. Stimulation was halted if there was evidence of arousal or awakenings and the entire block of data was rejected. Time permitted the inclusion of three blocks of data within each sleep stage. When more than three blocks were recorded, only the first three blocks were included in the analyses.

### Sleep Stage Scoring

Sleep staging was later confirmed on each 9.5-min sequence by two experienced sleep scorers using the American Academy of Medicine (AASM) task force criteria (Silber et al., 2007). Both scorers were blind to the real-time staging of the EEG. A 20 s epoch was employed rather than the usual 30 s to increase scoring precision. In cases of sleep stage change within the 9.5 min sequence or scoring ambiguity, the entire data within these sequences were rejected.

### ERP Analyses

**FIGURE 2:**
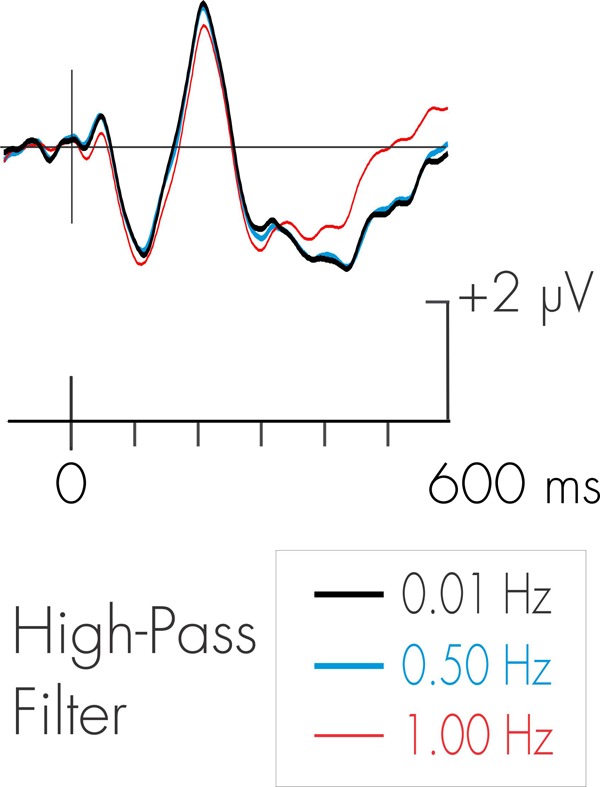
Effects of different high-pass filters on the P3a. ERP waveforms represent pilot data deviant-standard difference wave (environmental sound deviant) for 10 subjects in the waking state. As can be observed, there were minimal effects on the amplitude of the P3a with the use of various high-pass filters.

The data were then reconstructed using Brain Products’ Analyzer2 software. A 20 Hz (24 dB/octave) low-pass digital filter was subsequently applied to the continuous EEG data. The use of a high-pass filter is more problematic. This is because the amplitude of the background EEG during sleep is very high relative to the ERP signals of interest. This is particularly the case for low-frequency slow wave delta activity during NREM sleep. A means to attenuate the amplitude of the slow wave activity is through the use of high-pass filtering. The frequency spectrum of the P3a might, however, overlap with that of the low-frequency delta activity. Another late positive ERP component, the much-studied P3b (or “P300”), has been demonstrated to be distorted by excessive high-pass filtering (Duncan-Johnson and Donchin, 1979; Acunzo et al., 2012). Some compromise must therefore be exercised to avoid over filtering the ERP signals of interest, the DRN and P3a, while still attenuating the energy of the background delta activity. A waking pilot study was thus initially run with seven subjects to determine the effects of different high-pass filters on the P3a. Stimulus parameters were as described in the present study and subjects were asked to watch a silent video while ignoring the auditory sequence. Two different high-pass filters, 0.50 and 1.0 Hz were applied to the EEG and compared to the on-line 0.08 Hz high-pass filter. The 0.08 Hz filter would have minimal effect on the low-frequency delta activity in NREM sleep; the 1 Hz filter would markedly attenuate it. Following the filtering of the EEG, averaging procedures described below were applied to the data. The environmental sound deviant in the pilot data did elicit a large P3a. The effects of high-pass filtering on the pilot deviant-standard difference wave for the environmental sound deviant is illustrated in Figure 2. As can be observed, the 0.5 Hz high-pass filter had a minimal effect on the amplitude and latency of the P3a. The 1 Hz filter did have an effect on the waveform following the occurrence of the P3a. The 0.5 Hz high-pass filter was therefore employed in subsequent analyses of the data. Sabri and Campbell (2001) also noted that a 0.5 Hz filter had minimal effect on the morphology of the DRN. This is also apparent in Figure 2.

A vertical EOG channel was computed by subtracting activity recorded at the supra- and infra-orbital ridges of the left eye. A horizontal EOG channel was computed by subtracting activity recorded at the outer canthus of each eye. In the waking state and during stage REM, eye movements and blinks could cause artifact in the EEG recordings. Independent component analysis (Makeig et al., 1996; Chaumon et al., 2015) was used to identify the ocular activity that was statistically independent of the EEG activity. These artifacts were then partialled out of the EEG trace. The continuous data were subsequently reconstructed into discrete single trial 700 ms segments, beginning 100 ms before stimulus onset. A 0–50 ms post-stimulus baseline correction was applied to the waking data and all sleep stages because the pre-stimulus interval was not stable and varied between the waking state and sleep stages. It is possible that subjects might have been able to predict the onset of the stimulus during wakefulness thus affecting the pre-stimulus baseline. It would be expected that the first 50 ms following stimulus onset would mainly reflect stimulus-related sensory processing and thus should not have varied as a function of stimulus type. During wakefulness, segments in which EEG activity exceeded ±100 μV relative to the baseline were excluded from further analyses. No more than 5% of segments were rejected for any individual subject because eye movement had already been corrected. During stages N2, N3, and REM, this threshold was changed to ±200 μV to accommodate the large amplitude slow wave activity that is common to sleep. Because the amplitude of these slow waves would have been attenuated by the high-pass filter, fewer than 5% of segments were also rejected during sleep. There was no variation in the rejection of single segments across deviants or stages of sleep. The first 10 standard stimuli were excluded from the averaging procedure. The single trial segments were then sorted and averaged on the basis of state (waking, stage N2, stage N3, stage REM), stimulus type (standard and six deviants), and electrode site.

### Quantification and Statistical Analyses

The auditory deviant stimuli elicited a series of deflections that were not apparent in the standard ERP waveform. These deflections are best observed in a difference wave computed by subtracting, point-by-point, the standard from the deviant averaged waveforms at each electrode site. This process removes the commonalities in processing between the standard and the deviant, leaving only processing unique to the deviant. From this difference wave, the DRN and P3a were initially identified using the grand averaged data (the average of all subjects’ averages) within waking and stages N2, N3, REM. They were then quantified for each individual subject using the mean of all the data points within ±25 ms of the peak in amplitude that was identified in the grand average.

Previous studies have indicated that not all deviants will elicit a P3a during wakefulness. It was also possible that the P3a would be absent to some or all deviants during sleep. Thus, confidence intervals were computed to determine whether a deflection was significantly less or greater than the baseline setting (in the case of the DRN and P3a, respectively). The procedure was run on the Fz electrode site for the DRN and at Cz for the P3a where each tends to be at maximum amplitude. Because a directionality was predicted (negativity in the case of the DRN and positivity in the case of the P3a), one-tailed tests of significance (*p* < 0.05) were applied to the confidence intervals. To restrict the likelihood of chance findings, the negativity had to conform to the usual latency (100–250 ms) and scalp distribution (fronto-central maximum, inversion in polarity at the mastoids) of the DRN, while the positivity had to conform to the usual latency (180–350 ms) and scalp distribution (centro-frontal maximum) of the P3a.

Electrode sites were grouped into regions of interest (ROIs), to include nine electrode sites where the ERP components of interest were largest. The ROIs allowed for an analysis of an anterior–posterior and an inter-hemisphere factor. Specifically, for the anterior–posterior electrode factor, three electrodes for frontal (F3, Fz, F4), fronto-central (FC3, FCz, FC4), and central (C3, Cz, C4) sites were chosen for analysis. The DRN and P3a components were thus quantified at each of these sites within the latency range identified at Fz and Cz, respectively. For the inter-hemisphere factor, three electrodes for left (F3, FC3, C3), midline (Fz, FCz, Cz), and right (F4, FC4, C4) sites were chosen for analysis.

ANOVA procedures were then run to compare the amplitude of the DRN and P3a across waking and sleep stages. ANOVA testing was only run when a significant DRN or P3a was observed for a deviant during any of the sleep stages. Specific ANOVA procedures thus varied among the different deviants and will be described in the Section “Results.” Significant main effects and interactions were followed up with Fisher’s LSD *post hoc* testing. For all statistical analyses, a Geisser and Greenhouse (1958) correction was used when appropriate.

## Results

### Standard ERP

**FIGURE 3:**
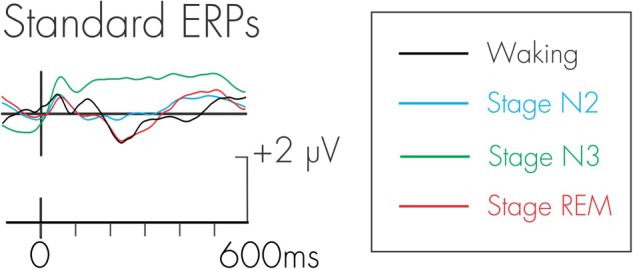
Grand average waveforms for the standard stimulus during waking, stage N2, stage N3, and stage REM.

Because the DRN and P3a were measured in a difference wave, an assumption is made that the processing of the standards is constant across all conditions. Any differences observed in the difference wave could therefore only be attributed to the additional processing of the deviant. This assumption was tested. Figure 3 depicts the ERPs elicited by the standard auditory stimuli during waking, stage N2, stage N3, and stage REM sleep. The amplitude of N1 and P2 were very small because of the fast rate of stimulus presentation. An ANOVA with repeated-measures on stage (waking, stage N2, stage N3, stage REM) was run at Cz to determine the effects of stage on the amplitudes of the standard N1 and P2. For the N1, there was a significant effect of stage, *F*(3, 45) = 15.42, MSE = 1.73, *p* < 0.0001, $\eta_{p}^{2}$ = 0.51. Fisher’s LSD revealed that the N1 during stage N3 was significantly smaller (more positive) than in waking, stage N2, and stage REM. As can be observed in Figure 3, this is due to the positive drift in the ERP waveform during stage N3. There was no significant difference in the amplitude of the N1 across waking, stage N2, and stage REM.

Similarly, for the P2, there was a significant effect of stage, *F*(3, 45) = 15.76, MSE = 2.64, *p* < 0.0001, $\eta_{p}^{2}$ = 0.51. Fisher’s LSD again revealed that the P2 during stage N3 was significantly larger than in waking, stage N2, and stage REM. There was no significant difference in the P2 between waking, stage N2, and stage REM.

An additional negativity at about 250 ms was also observed in the ERPs following the standard stimulus. There was a significant effect of stage for this negativity, *F*(3, 45) = 25.53, MSE = 3.73, *p* < 0.0001, $\eta_{p}^{2}$ = 0.63. Fisher’s LSD revealed that this negativity was significantly smaller (more positive) in stage N3 compared to waking, stage N2, and stage REM. The negativity was also significantly smaller in stage N2 compared to stage REM. There were no significant differences in its amplitude between waking and stage N2, and between waking and stage REM.

### DRN in the Waking State

**FIGURE 4:**
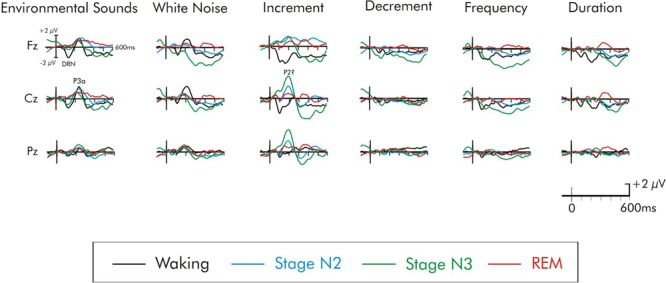
Grand average difference waveforms of the six deviants. ERPs shown at the Fz, Cz, and Pz electrode sites. The waveforms for the four different stages are superimposed for each deviant stimulus.

The difference waves for the various deviants across the waking and sleep states are illustrated in Figure 4. In the waking state, a negativity peaking at about 150 ms was observed following all deviants. It was maximum over frontal areas of the scalp and inverted in polarity at the mastoids, and thus corresponded to a DRN. Confidence interval testing in the waking state revealed that all deviants, except the decrement deviant, elicited a DRN significantly different from the zero-voltage baseline (*p* < 0.01 in all cases).

An ANOVA with repeated measures on deviant type (frequency, duration, decrement, increment, white noise, environmental sounds), frontality (frontal, fronto-central, central), and laterality (left, midline, right) was run on the waking DRN data. There was a significant main effect of deviant type, *F*(5, 75) = 5.76, MSE = 12.77, *p* < 0.0001, $\eta_{p}^{2}$ = 0.28. The DRN was largest to the increment deviant and smallest to the decrement deviant. The overall amplitude of the DRN was largest over frontal areas of the scalp, *F*(2, 30) = 12.24, MSE = 1.35, *p* < 0.0001, $\eta_{p}^{2}$ = 0.45 and was significantly reduced at central sites. The DRN was also largest over midline electrode sites, *F*(2, 30) = 6.33, MSE = 0.70, *p* < 0.01, $\eta_{p}^{2}$ = 0.30. Fisher’s LSD revealed no inter-hemispheric differences in the amplitude of the overall DRN between left and right areas of the scalp. Interactions involving deviant and electrode site were not significant (*F* < 1).

### DRN During Sleep

During stage N2 sleep, confidence interval testing indicated that none of the deviants elicited a DRN that was significantly different from the baseline (*p* > 0.05 in all cases). Surprisingly, during stage N3 sleep, the frequency and the decrement deviant did elicit a significant negativity during the time interval of the DRN (*p* < 0.05 in both cases). As can be observed in Figure 4, this could be due to an overall long-lasting negative drift that began prior to stimulus onset which is often observed during slow wave stage N3 sleep. The amplitude of the frequency DRN was nevertheless significantly larger during the waking state compared to stage N3, *t*(16) = 4.21, *p* < 0.01, but the decrement DRN did not significantly vary (*t* < 1) between the waking state and stage N3. All other deviants failed to elicit a significant DRN during stage N3. During REM sleep, none of the deviants elicited a significant DRN (*p* > 0.05 in all cases). A small negativity could be observed for the increment, but it was not significantly different from the baseline (*p* > 0.05).

### P3a in the Waking State

Not all of the deviants elicited a significant P3a in the waking state (Figure 4). In the difference waves, a large amplitude fronto-central maximum P3a was apparent only for the environmental sound and white noise deviants, peaking at about 240 and 215 ms, respectively. Confidence interval testing (Table 2) of the Cz data indicated that its amplitude was significantly different from baseline levels only for these deviants (*p* < 0.001 in both cases). An initial ANOVA with repeated measures on deviant type (white noise, environmental sounds), frontality (frontal, fronto-central, central), and laterality (left, midline, right) was run on the waking P3a data. The amplitude of the P3a to the environmental sounds and white noise deviants did not significantly differ (*F* < 1). The overall amplitude of these P3a data were largest over fronto-central areas of the scalp, *F*(2, 30) = 3.78, MSE = 0.60, *p* < 0.05, $\eta_{p}^{2}$ = 0.20, and significantly reduced at frontal sites. The P3a was largest over midline areas of the scalp, *F*(2, 30) = 15.46, MSE = 0.85, *p* < 0.0001, $\eta_{p}^{2}$ = 0.51. Fisher’s LSD again revealed no inter-hemispheric difference between left and right scalp regions. Interactions involving electrode site were not significant (*F* < 1).

**Table 2:** Mean amplitudes (SD in parentheses) at the Cz electrode site for the difference waves at the time interval of the P3a.

Deviant type	Stage	Amplitude (SD)	CI (95%)
Environmental sounds	Waking	1.89 (1.31)	[1.19, 2.59]
	Stage N2	1.71 (1.44)	[0.94, 2.48]
	Stage N3	1.09 (3.09)	[-0.55, 2.74]
	Stage REM	0.94 (1.34)	[0.22, 1.88]
White noise	Waking	2.06 (2.19)	[0.89, 3.23]
	Stage N2	0.81 (0.96)	[0.29, 1.32]
	Stage N3	0.33 (2.88)	[-1.21, 1.87]
	Stage REM	0.73 (1.48)	[-0.04, 1.52]

*Confidence intervals (95%) are also reported for the Cz electrode site.*

### P3a During Sleep

**FIGURE 5:**
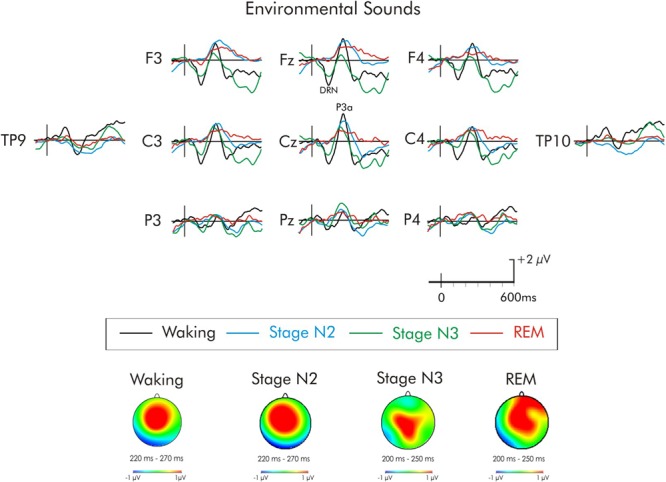
Grand average difference waveform for the environmental sounds at multiple scalp sites. The waveforms for the four different stages are superimposed. A significant P3a was observed in the waking state. A significant P3a-like positivity was also observed during stages N2 and REM. A subsequent positivity at about 320 ms can also be observed unique to REM sleep. Spline maps (bottom portion of Figure) are also presented for waking and stages N2, N3, and REM at the time interval of the P3a. During waking, stage N2, and stage REM, the P3a-like positivity was largest over centro-frontal regions, decreasing in amplitude over posterior and lateral regions. During stage N3, the distribution was more centro-parietal.

**FIGURE 6:**
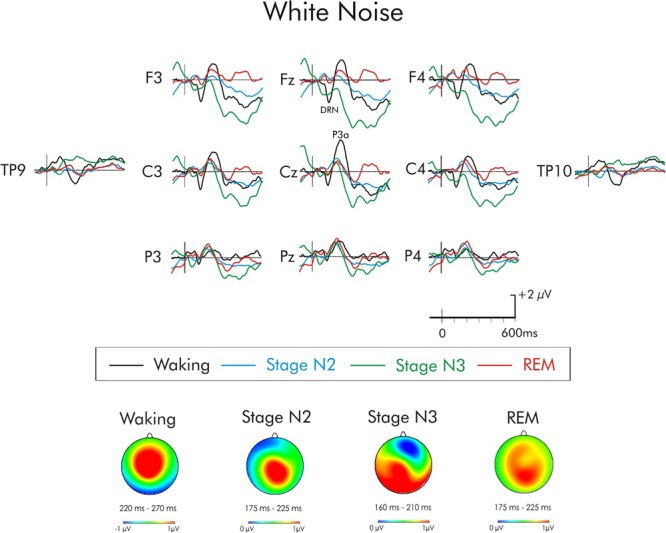
Grand average difference waveform for the white noise deviant at multiple scalp sites. The waveforms for the four different stages are superimposed. A significant P3a was observed in the waking state. A significant positivity was also observed during stage N2, it did not attain significant during stages N3 and REM. A subsequent positivity at about 400 ms can also be observed unique to REM sleep. Spline maps (bottom portion of Figure) are also presented for waking and stages N2, N3, and REM at the time interval of the P3a. During waking, the P3a was largest over centro-frontal areas of the scalp while during stages N2, N3, and REM it was largest over centro-parietal areas.

**FIGURE 7:**
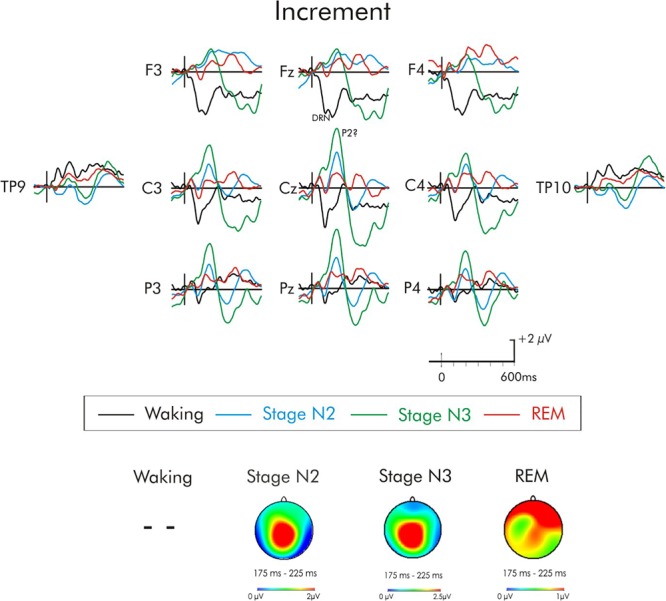
Grand average difference waveform for the increment deviant at multiple scalp sites. The waveforms for the four different stages are superimposed. A P3a was not elicited in the waking state. A significant positivity was observed during stages N2 and N3, it did not attain significance in REM. A subsequent positivity at about 350 ms can also be observed unique to REM sleep. Spline maps (bottom portion of Figure) are also presented for stages N2, N3, and REM at the time interval of the initial positivity. This positivity during stages N2 and N3 has a centro-parietal distribution, while during REM, this distribution was very broad, spanning from frontal to parietal regions.

Again, during sleep, not all of the deviants elicited a significant P3a-like positivity. For some deviants, a positivity at the time of the P3a was observed when it was not apparent in the waking state. Figures 5–7 illustrate the deviants at multiple scalp sites for which a P3a-like positivity was observed either during the waking or sleep states. During stage N2, confidence interval testing at Cz revealed that the environmental sound, white noise, and increment deviants elicited significant positivities at about 250, 190, and 200 ms, respectively (*p* < 0.01 in all cases). All other deviants failed to elicit a significant P3a during stage N2. The amplitude of the P3a was also compared in stage N2 occurring within the first and second halves of the night to determine possible time-of night-differences. The P3a did not significantly differ as function of time-of-night for either the environmental sound, white noise, and increment deviants (*p* > 0.05 in all cases). The stage N2 data were therefore collapsed across early and late halves of the night. During stage N3, only the increment deviant elicited a positivity at about 200 ms (*p* < 0.01). All other deviants, including the environmental sounds and white noise, failed to elicit a significant P3a-like positivity in stage N3 (*p* > 0.05). During stage REM, the environmental sounds again elicited a significant P3a-like positivity at about 220 ms (*p* < 0.01). A positivity was also observed following the white noise deviant, at about 190 ms, and increment deviant at about 200 ms. The positivity to both these deviants failed to attain significance (*p* > 0.05).

For the environmental sounds (Figure 5), an ANOVA with repeated measures on stage (waking, stages N2, N3, REM), frontality (frontal, fronto-central, central), and laterality (left, midline, right) was run. The amplitude of waking-P3a and the P3a-like positivities in during stages N2, N3, and REM were not significantly different (*F* < 1). The overall ANOVA revealed that the P3a was largest at fronto-central sites, *F*(2, 30) = 4.13, MSE = 0.79, *p* < 0.05, $\eta_{p}^{2}$ = 0.21. Spline maps (bottom portion of Figure 2) confirmed this for the waking, stage N2, and REM P3a-like positivities. During stage N3, however, the distribution was more central. Nonetheless, the interaction between stage × frontality just failed to reach significance, *F*(6, 90) = 1.95, MSE = 0.63, *p* = 0.08, $\eta_{p}^{2}$ = 0.11.

For the white noise deviant (Figure 6), the positivity observed during stages N2, N3, and REM occurred earlier (190 ms) than for the waking-P3a and its scalp distribution (bottom portion of Figure 3) was centro-parietally maximum, unlike the usual fronto-central P3a. Given the centro-parietal maximum distribution, the ROI analysis was adjusted with an ANOVA being run with repeated measures on stage (waking, stages N2, N3, REM) and electrode site (Fz, Cz, Pz). The amplitude of the waking P3a and the positivity in stages N2, N3, and REM were not significantly different, *F*(3, 45) = 1.83, MSE = 8.94, *p* = 0.16, $\eta_{p}^{2}$ = 0.11. The stage × electrode site interaction was, however, significant, *F*(6, 90) = 4.36, MSE = 0.92, *p* < 0.001, $\eta_{p}^{2}$ = 0.22. During waking and stage N2, the positivity was largest at Cz, while during stages N3 and REM, the positivity was largest at Pz.

In the case of the increment deviant (Figure 7), a large positivity was observed during stages N2 and N3. Similar to the positivity following white noise deviant during sleep, this positivity occurred earlier (200 ms) than the usual waking-P3a and its scalp distribution (bottom portion of Figure 4) was centro-parietally maximum, unlike the usual fronto-central P3a. An ANOVA was thus again run with repeated measures on stage (waking, stages N2, N3, REM) and electrode site (Fz, Cz, Pz). Overall, the positivity was significantly reduced during wakefulness compared to all sleep stages, *F*(3, 45) = 9.67, MSE = 10.10, *p* < 0.001, $\eta_{p}^{2}$ = 0.40. Additionally, Fisher’s LSD revealed the positivity in stage N3 was also significantly larger than during stage REM. The stage × electrode site interaction was also significant, *F*(6, 90) = 4.16, MSE = 1.24, *p* < 0.001, $\eta_{p}^{2}$ = 0.22. During stages N2 and REM, the positivity was largest at Cz, while during stage N3 it was largest at Pz. Spline maps of the positivity during stages N2 and N3 emphasize this centro-parietal distribution. During REM, this distribution was very broad, spanning from frontal to parietal regions.

### Late Positivity During REM

**FIGURE 8:**
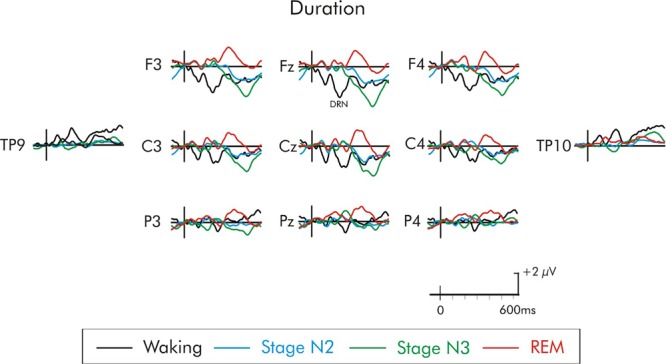
Grand average difference waveform for the duration deviant at multiple scalp sites. The waveforms for the four different stages are superimposed. A significant P3a was not elicited in the waking state or any of the sleep stages. A later positivity at about 350 ms can be observed unique to REM sleep.

A later positivity, at about 300–400 ms, occurring at about the same time as a positivity observed by Ruby et al. (2008), was also observed following some of the deviants, but only during REM sleep. This positivity was large over fronto-central areas of the scalp (Figures 5–8). When measured at Fz, it was significantly different from the baseline following the duration deviant (Figure 8), (*p* < 0.01) and the increment deviant (Figure 4), (*p* < 0.05). It did not reach statistical significance following either the environmental sound or white noise deviants (*p* > 0.05 in both cases). Previous studies have also reported a positivity at about this time, but with a parietal distribution (Bastuji et al., 1995; Cote and Campbell, 1999; Cote et al., 2001; Ruby et al., 2008). For this reason, it was measured at Fz, Cz and Pz. For the stage REM data, a two-way ANOVA with repeated-measure on deviant (environmental sounds, white noise, increment, and duration) and electrode site (Fz, Cz, Pz) was run. The amplitude of the positivity did not significantly differ among deviant types (*F* < 1). The overall amplitude of the positivity was slightly larger at Fz compared to Cz and Pz, although this difference was not significant (*F* < 1). The deviant type × electrode site interaction was also not significant (*F* < 1).

## Discussion

### DRN in Wakefulness and Sleep

The multi-feature paradigm proved to be successful in permitting a significant DRN to be elicited by all deviants in the waking state, except for the decrement. A small DRN was, in fact, observed following the presentation of the decrement, but it did not attain significance. The latency and scalp distribution of the waking DRNs essentially replicated the DRN findings observed by Tavakoli and Campbell (2016) and Tavakoli et al. (2018). In the present study, subjects were tested late in the evening compared to during the day in the Tavakoli and Campbell study. Thus, provided subjects are awake, the DRN appears to be well-preserved, even if sleep is very imminent. When subjects are no longer awake, during NREM (stages N2 and N3) and REM sleep, a discernible DRN was not apparent following most of the deviants. A significant negativity was observed in the difference wave following the frequency and decrement deviants during stage N3. These results should be interpreted with caution, however, as the processing of the standard ERP was significantly different during the time interval of the DRN during stage N3. Because of the difference in processing of the standard during stage N3, possible differences observed in the difference waves during this stage could be a result of the differences in processing of the standard. The lack of a DRN during NREM is in agreement with most previous studies that have used either frequency or intensity deviants (Nielsen-Bohlman et al., 1991; Winter et al., 1995; Loewy et al., 1996, 2000; Nashida et al., 2000). During REM, some studies have reported the presence of a DRN (Loewy et al., 1996; Atienza et al., 1997; Sabri and Campbell, 2005) while others have not (Sallinen et al., 1996; Loewy et al., 2000; Macdonald et al., 2008). Ruby et al. (2008) did report a significant MMN during both NREM and REM when a 30 ms deviant signaled a change from the duration of a 75 ms standard. In the present study, the duration deviant did not elicit a DRN. The duration of the standard and deviant stimuli (200 and 100 ms, respectively) were, however, much longer than those used by Ruby et al. (2008). It is possible that the sleeping brain can only detect brief and abrupt changes in stimulus duration. Additionally, the relative change in duration of the standard was larger in the Ruby et al. (2008) study than in the present one. Sabri et al. (2003) and Sabri and Campbell (2005) have also noted that a very large frequency change (1,000 Hz standard; 2,000 Hz deviant) elicited a DRN in stage N2 and REM but a smaller change (1,100 Hz) did not. The frequency deviant in the present study represented the same extent of change as the small frequency deviant used by Sabri et al. (2003; Sabri and Campbell, 2005); and also did not elicit a significant DRN during stage N2, N3, or REM.

It is also possible that the use of the multi-feature paradigm may account for the failure to observe a DRN during sleep. While the presentation of multiple deviants in the multi-feature paradigm permit the reliable recording of the DRN in the waking state, this may not be the case during sleep. The paradigm employed six different deviants each occurring quite rarely. Nevertheless, the overall probability of occurrence of deviants was 0.50, the standards and deviants being presented in an alternating pattern. Each different deviant does, however, share many features with the standard and as such, strengthens the sensory memory for the standard. Sabri and Campbell (2005) suggested that the failure to elicit an MMN during NREM sleep might be because of a rapidly fading sensory memory for the standard. In the case of the multi-feature paradigm, it is possible that the sensory memory for the standard is also poorly formed during sleep because it is only presented on 50% of trials. In studies employing oddball paradigms, the standard is presented on at least 80% of trials.

### P3a During Wakefulness

During wakefulness, a significant P3a was only observed following the environmental sounds (at 240 ms) and white noise deviants (at 215 ms). This replicates the results observed by Tavakoli and Campbell (2016). The amplitude of the P3a recorded during wakefulness in the present study was nevertheless somewhat reduced compared to that observed in the Tavakoli and Campbell study (about 1 μV). It is possible that this simply reflects between-group differences. A more likely explanation is that Tavakoli and Campbell (2016) tested subjects during the day and were thus presumably well-rested. In the present study, subjects were tested late in the evening when the demand for sleep was high. Similarly, in the Tavakoli et al. (2018) study, subjects were also tested late in the evening, and the waking P3a amplitudes are comparable to the present study.

Previous studies that have presented oddball paradigms have also observed a large P3a to environmental sound and white noise deviants compared to other pure tone deviants (Combs and Polich, 2006; Cahn and Polich, 2009; Berti, 2012; Frank et al., 2012; Wetzel et al., 2013). Tavakoli and Campbell (2016) and Tavakoli et al. (2018) also noted that the frequency, duration, decrement, and increment deviants did not elicit a P3a in the waking state. When a passive paradigm is used and subjects ignore the auditory sequence, it does appear that a large extent of change from the standard is required to elicit the P3a. On the other hand, when subjects are attending to the auditory sequence containing the deviant, much smaller deviants have been reported to elicit a P3a (Rinne et al., 2006; Grimm et al., 2008; Berti et al., 2013). The environmental sound and white noise deviants varied widely in terms of their frequency spectrum and stimulus energy (e.g., intensity) from the pure tone standards. All other deviants represented only a change in a single feature from the standard. Increment deviants in previous waking studies have repeatedly been shown to also elicit a P3a. The duration of the stimulus might again explain the discrepancies. In these studies, the duration of the increment deviants was very brief, typically 50 ms (Muller-Gass et al., 2006, 2007; Macdonald et al., 2008) compared to the much longer 200 ms used in the present study. Research has shown that the perceived intensity of a stimulus is directly affected by the duration of the stimulus (Zwislocki, 1960; Stévens and Hall, 1966). In other words, the perceived intensity of a stimulus increases as the duration of the stimulus is increased. However, in the case of oddball and multi-feature paradigms, the perceived intensity of both the deviant *and* standard increases. This may make the increment deviant to be less obtrusive.

### P3a During Sleep

No previous studies have used environmental sound deviants during all-night sleep. Remarkably, following the presentation of the environmental sounds, a positivity at the time of the waking P3a (230 ms) continued to be elicited throughout the entire night of sleep (i.e., during stages N2 and N3 of NREM sleep, and REM sleep). The scalp distribution of this P3a did not differ between waking and sleep states and the spline maps were very similar. As a result, there is little evidence to suggest that the intracranial sources of the P3a were different between the waking and sleep states. The positivity recorded to the environmental sound deviants during sleep thus appears to be a “true” P3a. Similarly, Tavakoli et al. (2018) also observed a significant P3a-like positivity during the sleep onset period during stage N1 and the first 30 min of stage N2. The environmental sounds, unlike any other deviant, differed on every trial, each containing a unique spectral content. By comparison, the spectral content of the other deviants was the same on each presentation. These environmental sounds are also more ecologically valid as most are sounds that are frequently experienced. These sounds could be sub-grouped from different categories of sound (musical instruments, birds, etc.). It is possible that certain highly salient and relevant sounds, such as human voices (Perrin et al., 1999; Pratt et al., 1999) or sounds having an emotional context (Pinheiro et al., 2016, 2017) might be processed more extensively than others. Unfortunately, it was not possible to average the various sub-categories of environmental sounds. Overall, the environmental sounds were presented on only about 8% of trials. As a result, an insufficient number of each sub-category of environmental sounds was presented to allow for a reduction of the background noise of the large amplitude EEG during sleep.

During both NREM and REM sleep, the increment and white noise deviants also elicited a positivity. This positivity, however, occurred around 190–200 ms, unusually early for a P3a. Tavakoli et al. (2018) also observed a larger positivity following the same increment deviant during the sleep onset period. Ruby et al. (2008) also observed a positivity in stages N2 and N3 that peaked earlier than the P3a in the waking state. In their case, the scalp distribution maps of the positivities in waking and sleep were similar. In the present study, the scalp distributions were different. For both deviants, it was maximum over centro-parietal regions of the scalp during stages N2 and N3 compared to the fronto-central scalp distribution of the P3a. During REM, the distribution was much broader. ERP components that have different scalp distributions must have different intra-cranial sources (Picton et al., 1995). It is, therefore, unlikely that the positivity to the increment and white noise deviants reflect the same P3a source during the waking and sleeping states. Additionally, the increment deviant did not elicit a P3a in the waking state. Some studies have reported a P3a-like positivity during stage REM following the presentation of a particularly large increase in stimulus intensity (Cote and Campbell, 1999; Macdonald et al., 2008) but this increment deviant also elicited large P3a in wakefulness. It is possible that these positivities reflect the earlier P2. The peak latency and scalp distribution of this positivity following both increment and white noise is more consistent with the more usual posterior scalp distribution of the P2 than the more anterior P3a. Many sleep studies have observed that the amplitude of the P2 may increase during NREM sleep (Nielsen-Bohlman et al., 1991; Winter et al., 1995; Nittono et al., 2001; Crowley et al., 2002; Macdonald et al., 2008; Campbell and Muller-Gass, 2011). In the present study, the amplitude of the increment positivity was much also much larger during NREM sleep than in wakefulness. In their review, Crowley and Colrain (2004) have suggested that the amplitude of P2 reflects an inhibition of processing. Thus, the appearance of a large P2 during NREM reflects a need for the protection of sleep. The P2 was larger in stage N3 than N2. This is also consistent with a greater for sleep to remain undisturbed during deep “slow wave sleep” (stage N3) than in the lighter stage N2.

### Late Positivity During REM

A later small amplitude positivity, at about 300–400 ms, was also observed but was unique to REM sleep. What was especially surprising was that it was elicited by many different deviants and its amplitude did not significantly differ among them. It had a fronto-central distribution. It is possible that it simply reflects residual background noise. However, it was found to be consistently elicited at about the same latency and to have a similar scalp topography across multiple deviants. Moreover, other studies have also reported a late positivity (around 300–500 ms) during REM (Bastuji et al., 1995; Cote and Campbell, 1999; Perrin et al., 1999; Cote et al., 2001; Ruby et al., 2008). In these studies, the scalp distribution was different, the late positivity having a distinctly centro-parietal maximum. This scalp distribution is consistent with the much-studied P3b.

It is possible that the early and late positivities during stage REM reflect a delayed P2 and P3a. The early positivity did have a centro-frontal scalp distribution, consistent with an actual P3a. As mentioned above, the scalp distribution of the P2 is more posterior. Escera et al. (1998) observed that their waking P3a to environmental sounds had two distinct subcomponents. The early portion of the P3a, peaking at around 230 ms, had a centrally dominant scalp distribution. The late portion of the P3a, on the other hand, peaked at around 330 ms and displayed a frontally maximum scalp distribution. The late positivity observed during stage REM in the present study appears to be similar to the late portion of the P3a described by Escera et al. Nevertheless, it is surprising that similar early and late subcomponents were not observed in the waking state. The functional significance of late positivity in the present study occurring only in REM sleep is thus difficult to interpret.

### Disassociation of the DRN and P3a

A DRN was not observed during either NREM or REM sleep, yet a P3a-like positivity was still elicited following some deviants. Based on the classic Näätänen model, the probability of eliciting a P3a is expected to decrease as the amplitude of the MMN/DRN is reduced. More recent studies have now suggested that the amplitude of the MMN/DRN and P3a are not necessarily linked (Sussman et al., 2003; Fischer et al., 2008; Horváth et al., 2008). Therefore, a deviant might elicit a P3a in the absence of an MMN. Näätänen also notes that other factors may affect the P3a, suggesting that the threshold for its elicitation is flexible. It is possible that during sleep the threshold to elicit the P3a is significantly lowered in order to alert the individual to potentially highly relevant information in the environment. Thus, even though the amplitude of the MMN/DRN is reduced, the P3a may still be elicited.

## Conclusion

The purpose of this study was to determine whether potentially highly relevant stimulus change can intrude into conscious awareness during all-night natural sleep using an auditory multi-feature paradigm. In the present study, a fronto-central maximum P3a was passively elicited in the waking state by environmental sound deviants. Importantly, the environmental sounds continued to elicit a similar positivity occurring at about the same time during both NREM and REM sleep. Subjects rarely were awakened by the auditory stimuli, even by the environmental sounds that did elicit a large P3a. Thus, while these stimuli might be considered to be potentially highly relevant and meriting additional processing as reflected by the presence of the P3a, in the end, they turn out to be not so critical as to warrant sleep to be reversed.

Other deviants failed to elicit a definitive P3a during sleep. Another earlier positivity having a different scalp distribution than the P3a was elicited by the white noise and increment deviants during stages N2, N3, and REM, although it did not attain significance in REM following either deviant. This positivity may be an earlier P2 often reported to increase in amplitude during sleep. The environmental sounds, white noise, increment, and duration deviants also elicited a later fronto-central maximum positivity unique to REM which has not been reported in previous studies. What this late positivity reflects is largely unknown. Again, awakening during REM sleep was relatively rare.

It should be noted that auditory stimuli may trigger evoked K-Complexes during NREM sleep which may affect the shape of the ERP waveform (Czisch et al., 2009). The presence of evoked K-Complexes were not accounted for in the present study. Nonetheless, K-Complexes are only elicited when stimuli are presented very slowly (>10 s; Bastien and Campbell, 1994). Thus, very few K-Complexes would have been elicited, even by the deviants that did elicit a P3a. Moreover, the large negativity associated with the K-Complex (the N550) occurs well after the P3a and, therefore, should not affect its morphology.

The current study also has implications for other unconscious states such as general anesthesia and coma. Researchers are currently employing ERP techniques to probe the extent of information processing and consciousness in patients who may be covertly conscious (for reviews, see Gawryluk et al., 2010; Harrison and Connolly, 2013; Morlet and Fischer, 2014). The multi-feature paradigm may be a useful tool in these clinical and applied studies.

## Ethics Statement

The study was approved by the University of Ottawa’s Health Sciences and Science Research Ethics Board. Written informed consent was obtained prior to the start of the study and subjects received an honorarium for their participation. The study was conducted according to the Canadian Tri-Council guidelines (Medical, Natural, and Social Sciences) on ethical conduct involving human subjects. These guidelines are similar to those used with the Declaration of Helsinki.

## Author Contributions

PT, KC, AB, and AD contributed to the rationale and the design of the study and read and approved the final manuscript. The manuscript was written by PT. PT and AD assisted with the collection and analysis of the EEG data. KC, AD, and AB provided feedback and revisions on written drafts of the manuscript.

## Conflict of Interest Statement

The authors declare that the research was conducted in the absence of any commercial or financial relationships that could be construed as a potential conflict of interest.
